# Individual, family and environmental factors associated with pediatric excess weight in Spain: a cross-sectional study

**DOI:** 10.1186/1471-2431-14-3

**Published:** 2014-01-08

**Authors:** José-Juan Sánchez-Cruz, Ingrid de Ruiter, José J Jiménez-Moleón

**Affiliations:** 1 Andalusian School of Public Health (EASP), Granada, Spain; 2 Department of Preventive Medicine and Public Health, University of Granada, Granada, Spain; 3 CIBER of Epidemiology and Public Health (CIBERESP), Granada, Spain

**Keywords:** Overweight, Child, Adolescent, Risk factors, Physical activity, Breakfast

## Abstract

**Background:**

There is a growing worldwide trend of obesity in children. Identifying the causes and modifiable factors associated with child obesity is important in order to design effective public health strategies.

Our objective was to provide empirical evidence of the association that some individual and environmental factors may have with child excess weight.

**Method:**

A cross-sectional study was performed using multi-stage probability sampling of 978 Spanish children aged between 8 and 17 years, with objectively measured height and weight, along with other individual, family and neighborhood variables. Crude and adjusted odds ratios were calculated.

**Results:**

In 2012, 4 in 10 children were either overweight or obese with a higher prevalence amongst males and in the 8–12 year age group. Child obesity was associated negatively with the socio-economic status of the adult responsible for the child’s diet, OR 0.78 (CI95% 0.59–1.00), girls OR 0.75 (CI95% 0.57–0.99), older age of the child (0.41; CI95% 0.31–0.55), daily breakfast (OR 0.59; p = 0.028) and half an hour or more of physical activity every day. No association was found for neighborhood variables relating to perceived neighborhood quality and safety.

**Conclusion:**

This study identifies potential modifiable factors such as physical activity, daily breakfast and caregiver education as areas for public health policies. To be successful, an intervention should take into account both individual and family factors when designing prevention strategies to combat the worldwide epidemic of child excess weight.

## Background

The World Health Organization (WHO) defines obesity as a disease, a complex condition with physical, social and psychological dimensions, with serious health and economic consequences [1].

In the U.S. the 2009–2010 prevalence of overweight and obesity is 31.8% for children aged 2 to 19 years [2]. Europe estimates 20% of children and adolescence to be overweight, with one third of these obese and the annual rate of increase in this prevalence is growing [3]. In Spain, the 2011 National Health Survey of 5495 children reported a combined prevalence of overweight and obesity of 29.1% in boys aged 2 to 17 years and 26.5% in girls of the same age; slightly higher than previous National Health Survey outcomes [4].

Child obesity results in both immediate as well as long term health consequences as risk profiles track into adulthood [3,5,6]. These include social and psychological issues as well as orthopedic problems, type 2 diabetes, hypertension, sleep apnea, metabolic syndrome and lower quality of life [3,7-11]. Identifying the causes and modifiable factors associated with child obesity is important to be able to design effective public health strategies to reverse the current obesity trends.

Multiple factors, including genetic, environmental, cultural and socio-economic status may influence corporal weight [12-18]. Researchers of child and adolescent obesity have mainly focused on individual factors such as gender, socio-economic position, physical activity, sedentary habits, nutrition and sleep duration [12,13,19]. Evidence also suggests that environmental and family factors influence adopted habits, particularly in children [14-16,20,21]. The neighborhood environment can include both physical aspects, which create opportunities or barriers for obesogenic behaviors, and social aspects of perceived safety or facility availability [22,23]. Additionally, in children and adolescents the changing level of autonomy with age combined with parental perception of neighborhood characteristics may influence obesity related behaviors. Positive correlations between parent-reported neighborhood characteristics and child physical activity have been identified in other studies [23]. Nonetheless, the use of environmental factors in children may be difficult due to the ecological characteristics of this type of variable with a high probability of misclassification bias, difficulty to separate familial and environmental factors [15], as well as constraints in establishing causal relationships between environmental factors and child obesity. Scientific literature provides partial, incomplete, sometimes contradictory and, therefore, inconclusive findings regarding the association of many of the individual and environmental factors on obesity. There is a need for new research that combines these different types of factors, in particular with the addition of family and environmental variables. This study aims to provide empirical evidence of the association that some individual, family and environmental factors may have on excess body weight during childhood and adolescence.

## Methods

### Study design and population

A cross-sectional observational study was carried out using probability sampling of the study population. The study population consisted of children and adolescents, of both sexes, between the ages of 8 and 17 years inclusive, resident in family households in peninsular Spain. Data were collected during April and May in 2012.

The probabilistic sample was based on a multistage clustered and stratified sample. Primary sampling units (municipalities) and secondary units (census groups) were selected through a probability proportional to size (PPS) method. Tertiary units (households) and individual units were selected using a combination of random pathways and quotas for sex and age. Population strata were formed by the intersection of the 15 mainland regions with municipality population size divided into 5 categories: (1) less than or equal to 2000 inhabitants; (2) 2001 to 10 000; (3) 10 001 to 50 000; (4) 50 001 to 200 000; and (5) more than 200 000 inhabitants. The selected sample was proportional to the size of the strata. The distribution of the two age groups in the sample population (8–12 years and 13–17 years) was equal to their proportions in the population.

### Measures and selected variables

Weight and height measurements were taken in the presence of the adult responsible for the child’s diet, who was also asked to complete socio-demographic questions via computer-assisted personal interview, see Additional file 1. Anthropometric measures of the different household members were measured using a scale and height rod and followed a set measuring protocol. The specific models used were: a) Scale – Tefal PP1027 A9, and b) Height rod: 5002.01.001 Soehnle professional. The child was placed in the standing position, without shoes, with hips and shoulders perpendicular to the central axis of the body, heels firmly planted on the ground, knees close together and extended, relaxed arms, and head in the Frankfurt plane. Body weight was determined through a digital anthropometric scale graded from 0 to 150 kg with a resolution of 0.05 kg. The body mass index (BMI) was calculated by the quotient of body mass in kg by height in meters squared (m^2^), and subsequently overweight and obesity were defined according to the World Health Organization criteria [24]. Excess weight was defined as the presence of overweight or obesity in the child or adolescent at the moment of recruitment.

The questions and response scale used in the computer-assisted personal interview are part of the standardized questionnaire used in the Andalusian and National Health surveys. The questionnaire used in our study was also initially tested on a sample of 50 people of the target population.

The variables considered in this study, with their initial categorization, were: a) age group of child: 8–12 years (REF), 13–17 years; b) sex of child: male (REF), female; c) population category of municipality: less than or equal to 2000 inhabitants, 2001 to 10 000, 10 001 to 50 000, 50 001 to 200 000, and more than 200 000 inhabitants; d) Education level of the adult responsible for the child’s diet: Primary (REF), Secondary or University level studies; e) Employment status of the adult responsible for the child’s diet: Employed, Unemployed but previously employed, Looking for first employment, Retired (worked previously), Housewife, Student, Disability, Permanent Disability, Other; f) Occupation of the adult responsible for the food of the children according to the national classification of occupations 2001 (CON-11); g) Walking: less than 30 min per day (REF), 30 min or more per day; h) Sleep duration: less than 9 hours per night (REF), 9 hours or more per night; i) Variables related to dietary habits, including: daily breakfast, daily freshly-squeezed orange juice, daily Yoghurt; j) TV watching: watches TV every day, does not watch TV every day; k) Perception of neighborhood quality: Good/Very Good, Average/Poor/Very Poor (REF); l) Perception of Neighborhood Safety: Good/Very Good (REF), Average/Poor/Very Poor; m) Caregiver perception of Excess Weight: Excess weight is not detrimental to health, Is detrimental but not as much as is alleged by doctors or the media, Is detrimental for health. Some variables were subsequently reclassified, as shown in Table 1, due to the low number of observations in some categories or due to similar behavior with respect to the dependent variable. Maternal and paternal ages were considered as continuous variables.

**Table 1:** Descriptive characteristics of survey population

		**n, mean (SD)**^**1**^
		**n (%)**^**2**^
**Variables related to the child**		
**Age**		976, 11.99 (2.94)
	8-12	534, 9.64 (1.38)
	13-17	442, 14.82 (1.43)
**Sex**	Male	490 (50.20)
	Female	486 (49.80)
**Breakfast daily**		
	4days or less per week	89 (9.1)
	5 or more days per week	887 (90.9)
**Freshly squeezed orange juice**		
	7 days per week	105 (11.2)
	Less than 7 days per week	832 (88.8)
**Daily yoghurt**		
	7 days per week	99 (10.6)
	Less than 7 days per week	839 (89.4)
**TV daily**		
	Watches TV everyday	459 (47.2)
	Does not watch TV everyday	514 (52.8)
**Physical activity**		
< 30 min per day		527 (54)
30 min or more per day		446 (46)
**Sleep duration**		
Less than 9 hrs/day		399 (40.9)
9 or more hrs/day		577 (59.1)
**Variables related to the family**		
**Maternal age**		950, 40.76 (6.06)
**Paternal age**		798, 43.43 (6.19)
**Adult responsible for child’s diet**		
	Father	114 (11.7)
	Mother	826 (84.6)
	Others	36 (3.6)
**Academic level of adult responsible for food**		
	Primary	538 (57.2)
	Secondary/University	402 (42.8)
**Occupation of adult responsible for food**		
	Manager/Professional	70 (11.8)
	Unskilled worker/other	523 (88.2)
**Employment status of adult responsible for food**		
	Working	476 (52.2)
	Unemployed	135 (14.8)
	Housewife	301 (33.0)
**Caregiver perceptions of excess weight**		
	Excess weight is not detrimental to health	11 (1.1)
	Is detrimental, but not as much as is said by doctors or in the media	19 (1.9)
	Is detrimental for health	945 (96.8)
**Variables related to the environment**		
**Neighbourhood quality**		
	Good/Very Good	795 (81.7)
	Average/Poor/Very Poor	178 (18.3)
**Neighbourhood safety**		
	Good/Very Good	819 (84.0)
	Average	129 (13.2)
	Poor/Very Poor	27 (2.8)

^1^n, mean (SD): Sample size, Mean (Standard Deviation).

^2^n (%): Absolute frequency (Percentage).

### Data analyses

For descriptive statistics, the mean and standard deviation were calculated for continuous variables. For categorical variables, percentage distributions are shown. Comparison of proportions was carried out using the chi-squared statistic if its conditions were met, and if the conditions were not met the Fisher exact test was used. In order to jointly analyze the relationship of the considered independent variable with respect to excess weight, a logistic regression model was applied. Possible factors associated with excess weight were included in this model and their odds ratios obtained. Maternal age was considered as continuous when modeling the data, as its relationship with the log of excess weight prevalence was approximately linear.

First, a logistic regression model of excess weight with respect to the child’s sex and age was fitted. Next, a new variable was added successively in each step (using the forward method of introducing variables manually). The variables selected to introduce into the models were chosen according to epidemiological and statistical criteria. The effect of each exploratory variable in the model and its significance was studied. If the variable improved the model fit and adequacy (based on the likelihood ratio criteria and the significance of the parameter) it was kept for the next step; otherwise, the variable was excluded. Different models were fitted with respect to the factors related to the family and physical environment. The model was checked for pair-wise interaction between covariates. Interactions with the sex and age of the child were considered. Potential confounding covariates were studied using a change of significance of the parameters in the model or a change of 30% of its value [25]. Once the model was fitted to the data, the goodness of fit of the model was assessed by the Hosmer-Lemeshow test. SPSS statistical package version 18 was used to perform all analyses.

### Ethics statement

Ethics approval was obtained from the Research and Ethics Committee of the Andalusia School of Public Health (Regional Ministry of Health, Regional Government of Andalusia), with assurance of the anonymity of individual data in accordance with the requirements of Spanish law. Verbal consent was obtained from parents or legal guardians as a pre-requisite to collecting information. Consent procedure required an explanation of the research project, what it consisted of and the type of data being collected.

## Results

A total of 978 children were included in the analyses and an overall participation rate of 80% was achieved. The overall prevalence of overweight and obesity was 38.6% (CI95% 35.5 – 41.6%). Baseline characteristics of the study population are shown in Table 1. The mean age of the group was 12 years old (SD 2.94) and 50.2% of the whole sample were male. The mother was the adult responsible for the child’s diet in the majority of cases (84.6%) and over 80% of the adults responsible for diet perceived neighborhood safety and quality as either good or very good. The majority (90%) of children ate breakfast at least 5 days per week. Just over half of children exercised at least 30 minutes per day, and around 47% also watched television daily. With respect to sleeping hours, 41% slept less than 9 hours per day on average.

The crude odds ratios are shown in Table 2 and the adjusted odds ratios (OR) in Table 3. The statistically significant associations found in the crude models held in the adjusted model for age, sex, walking, and for the child having daily breakfast. In both the crude and adjusted analyses the education level of the adult responsible for the child’s diet bordered on statistical significance (p < 0.10). Regarding the child’s characteristics, females were found to have a 25% lower risk of excess of weight compared with males. We observed a higher risk of overweight and obesity in younger children (8–12 years) compared with adolescents (13–17 years): the risk was 59% lower in this last group compared with children aged 8–12, adjusted OR 0.41 (CI 95%, 0.31 – 0.55). A lower risk was also found if the child walked at least 30 minutes per day, adjusted OR 0.73 (CI 95%, 0.56 – 0.96). Children that ate daily breakfast were observed to be less likely to have excess weight, adjusted OR 0.59 (CI 95%, 0.36 – 0.94). With respect to the characteristics of the adult responsible for the child’s diet, we found a trend towards a lower risk of excess weight with a higher level of education of the adult responsible, adjusted OR 0.78 (CI 95%, 0.59-1.03). In our study, variables related to the perceived environment were not associated with youth excess weight in either the crude or in the adjusted analyses, as can be seen in Table 2.

**Table 2:** Factors associated with excess weight in Spanish youth aged 8-17

		**Crude analysis**		
		**OR**	**95% CI**	**p**
**Variables related to the child**				
**Sex**	Male	1	Ref.	
	Female	0.79	(0.61 – 1.02)	0.071
**Age**	8-12 years	1	Ref.	
	13-17 years	0.86	(0.82 – 0.90)	**0.000**
**Physical Activity**	<30 min/day	1	Ref.	
	≥30 min/day	0.73	(0.56 – 0.95)	**0.019**
**Sleep duration**	<9 hrs/night	1	Ref.	
	≥9 hrs/night	1.06	(0.81 – 1.38)	0.676
**Breakfast daily**	No	1	Ref.	
	Yes	1.47	(0.95 – 2.28)	0.083
**Related to the family**				
**Age of mother**	Per year Increase	0.97	(0.96 – 0.98)	**0.000**
**Age of father**	Per year Increase	1.00	(0.99 – 1.00)	0.727
**Level of education of adult responsible for food**	Primary	1	Ref.	
	Secondary/University	0.77	(0.59 – 1.00)	0.051
**Occupation of adult responsible for food**	Professional/ Manager	1	Ref.	
	Unskilled worker/other	0.54	(0.31 – 0.94)	**0.028**
**Employment status of adult responsible for food**	Self-employed/ Housewife	1	Ref.	
	Unemployed	1.32	(0.87 – 2.01)	0.191
	Employed	1.35	(1.00 – 1.82)	0.052
**Related to neighbourhood**				
**Perception of neighbourhood security**	Good or very good	1	Ref.	
	Average	1.22	(0.84 – 1.78)	0.300
	Poor or very poor	1.13	(0.52 – 2.46)	0.761
**Perception of neighbourhood quality**	Very poor, poor or average	1	Ref.	
	Good or very good	1.04	(0.74 – 1.45)	0.836
**Parental view on child obesity**	Obesity is bad for health	1	Ref.	
	Obesity is not bad for health	1.85	0.90, 3.83	0.099

**Table 3:** Factors associated with excess weight in Spanish youth aged 8–17 - Multivariate Analysis

		**Adjusted analysis**		
		**OR**	**95% CI**	**P**
**Variables related to the child**				
**Sex**	Male	1	Ref.	
	Female	0.75	(0.57 – 0.99)	0.043
**Age**	8-12 years	1	Ref.	
	13-17 years	0.41	(0.31 – 0.55)	0.000
**Physical activity**	<30 min/day	1	Ref.	
	≥30 min/day	0.73	(0.56 – 0.96)	0.027
**Breakfast daily**	No	1	Ref.	
	Yes	0.59	(0.36 – 0.94)	0.028
**Related to the family**				
**Level of education of adult responsible for food**	Primary	1	Ref.	
	Secondary/ University	0.78	(0.59 – 1.03)	0.082

Variables in the crude analysis that were found to be not significant in the multivariate analysis are not included in Table 3.

## Discussion

This study provides information on factors associated with the prevalence of excess weight among children and adolescents aged 8–17 in Spain. In 2012, approximately 4 out of every 10 children and adolescents were overweight or obese with a higher prevalence amongst males and those aged 8–12 years. Child obesity was also shown to be associated with the sex of the child, the education level of the adult responsible for the child’s diet, the level of physical activity of the child as measured by time walking per day, and having breakfast daily. For this reason, both individual and family factors should be taken into account in the fight against the worldwide epidemic of child excess weight.

Consistent with scientific literature in this area, our findings show that the risk of excess weight is lower in females than males. This result is consistent with observational studies in Europe, Japan and the USA [2,26-28]. As has been previously commented on in literature, differences in sexes could potentially be due to a variety of influencing factors such as hormonal differences during and post- puberty, cultural gender constructs or differing influences of environmental or familial variables [29]. However, the association between sex and excess weight persists in our study after adjusting for potential confounding factors related to individual, environmental and familial variables. These differences could be explained by the role that non-modifiable variables, such as genetic and hormonal factors, play in the weight of a person.

In this study, older children were found to have a lower risk of excess weight than younger children. This finding could potentially be explained by an age-effect or could indicate that the problem is getting worse over time and will grow in the future if we do not act expediently [30]. The observed difference between age-groups close in time is worrying and warrants further investigation.

Independently of the age and sex of the child, the role that modifiable factors such as diet and physical activity play in the obesity epidemic are clear and well established. However, most interventions have focused mainly on the role of diet rather than on the role of physical activity. Not having breakfast has been classically identified as a risk factor for excess weight in childhood [17,31] and many interventions have been made to combat this factor and as such, according to our results, the problem currently affects less than 10% of Spanish children. However, it seems that so far physical activity has not been given the attention it deserves. Our findings show that children carrying out physical activity on a regular basis are less prone to suffer from excess weight than those with sedentary habits, independent of sex or educational level of the adult responsible for diet. Physical activity is considered an important factor in energy balance and a growing body of evidence suggests that reduced daily physical activity is a main cause of the worldwide increase in youth obesity and overweight [1,32,33]. Physical activity should be promoted as part of a healthy lifestyle to prevent excess weight gain and this promotion should begin at an early age. However, despite efforts being made, success is limited and variable [34,35]. Perhaps a global and integrated approximation to the problem is necessary, considering not only the child but also the family and the environment where the child lives.

The behavior of children depends largely on the family environment in which they grow and we cannot analyze a child’s diet and level of physical activity without considering the family environment. In this sense, a higher level of parental education is less associated with overweight and obesity in the offspring and may be related to differing lifestyle choices such as diet quality and act in this way [17,31]. Although, considering the design and information of our study, we were not able to analyze these hypotheses. The IDEFICS consortium, based on data from a cross-sectional baseline survey of a prospective cohort aged 2 to 9 years in eight European countries, shows that the intakes of vegetables, fruits, pasta/noodles/rice, wholegrain bread and water increased as educational level increased; while intakes of fried potatoes, fried meat and fish, fast food, sugared beverages, snacks/desserts and chocolate/nut-based spread increased as educational level decreased [17]. Further study is required to clarify this relationship and investigate the underlying mechanisms.

When the factors associated with child excess weight are analyzed, we can clearly identify two types of factors: a group of factors that depends directly on the child and their behavior, and another group of factors related to the family environment. The frontier between both groups of factors is very difficult to establish, but if we want to be successful in the control and prevention of child excess weight we should consider both groups in the design of adequate interventions. Perhaps family factors have not been playing the real role that they should in child obesity prevention campaigns.

Along with individual and familial factors, we should consider the role that environmental factors may play in facilitating the adoption of healthy lifestyles. Certain environmental factors are widely considered as relevant for the development and prevention of obesity, influencing directly or indirectly the motivation of children to engage in physical activity. They may also influence diet quantity and quality, due to the availability of opportunities and places for the consumption of healthy or non-healthy foods [18,22,36]. Our findings showed no association of the measured environmental factors with excess weight in youth. There are several reasons that should be taken into account to explain these differences: 1) It is possible that the environmental variables considered in our study were measured in a subjective and perceived manner with a potential distortion from reality (if measured objectively). This could create difficulty in finding statistically significant differences compared with objective measures. For example, Bodor JN et al. described a high risk of obesity associated with fast food restaurants and convenience stores, but had not considered neighborhood quality and safety variables [36]; 2) Studies where they relate obesity to physical environmental factors usually work with adult population samples [22,36]; 3) The magnitude of the associations found between obesity and environmental characteristics are usually very weak, 1.01 (1.00 – 1.02) for fast food restaurants and obesity in Bodor’s study [36]; and 4) Alternatively, environmental factors could indirectly influence obesity-related behavior through individual and familial variables, which can be very important in children [23]. Whatever the reason is for why environmental factors were not significant in this study is beyond the scope of this research.

As potential limitation of our study we should cite: 1) Its observational nature and the weakness associated with any cross-sectional study in that no temporal relationship or direction of association can be determined. One obvious drawback in this type of epidemiologic study is possible reverse causation or common upstream cause. Cross-sectional associations may reflect the combined intervention of the true effects of a particular factor as well as artificial effects due to reverse causation and confounding by other variables. The absence of association found between variables related to diet and weight in our study could be explained by this and it can’t be forgotten that diet is one of the first things that people modify when wanting to lose weight; 2) Another limitation to be considered is the subjective nature of interviewees’ perceptions of their neighborhood and level of physical activity, as previously discussed. However, we can also consider that the perception of neighborhood security may be more important in level of physical activity than objective neighborhood security; 3) We should also keep in mind that marginal social classes are not included in health surveys. In addition, children who belong to the lower (but non marginal) social classes enjoy great protection due to the public policies of the Spanish welfare state, and this can attenuate the observed associations between socio-economic status and excess weight. As advantages of our study we should highlight that: 1) Our sample is representative of Spanish children 8–17 years. The multi-stage probability sampling method used resulted in a sample that was representative of the target population, meaning that the results can be extrapolated to a greater population; 2) While many studies use subjective measures for child weight, this study used objective measures for weight, height and BMI outcomes; 3) We tried to integrate the role of individual and social factors on the risk of excess of weight in children, unlike other studies that are based on only individual variables; 4) Missing data were minimal and were not different from data of the included participants.

## Conclusions

The results in this paper provide relevant information to be considered when developing public health policies, professional care in the area of childhood overweight and obesity and further research. In our study we identify as areas for public health policies and further research potentially modifiable factors such as physical activity and caregiver education. If we want to be successful, an adequate intervention for the control of the obesity epidemic in children should not forget to act on both the child and his social context.

## Competing interests

The authors declare no conflict of interests.

## Authors’ contributions

JJSC is the main researcher of the project number PI10/02018, he participated in the design of the study and statistical analysis of the data and the discussion of the results. IdR has collaborated in the analysis, discussion of the results and preparation of the initial draft. JJJM contributed to the analysis and discussion of the results. All authors were involved in writing the finished paper and had final approval of the submitted and published versions.

## Pre-publication history

The pre-publication history for this paper can be accessed here:

http://www.biomedcentral.com/1471-2431/14/3/prepub

## Supplementary Material

Additional file 1 **File name: cuestionario obesidad infantil BMC Pediatrics.pdf. File type: Acrobat file.** Title of dataset: Estudio sobre obesidad infantil y juvenil. Description: Questionnaire used in this study to collect socio-demographic information on study participants. Click here for file
