# Supplementary material for: Individual, family and environmental factors associated with pediatric excess weight in Spain: a cross-sectional study
Source: BMC Pediatr. 2014 Jan 8;14:3. doi: 10.1186/1471-2431-14-3 (PMC3897889; doi:10.1186/1471-2431-14-3)
Supplement: Additional file 1 — File name: cuestionario obesidad infantil BMC Pediatrics.pdf. File type: Acrobat file. Title of dataset: Estudio sobre obesidad infantil y juvenil. Description: Questionnaire used in this study to collect socio-demographic information on study participants. [file 1471-2431-14-3-S1.docx]

***[Translation of questionnaire used during this study. Original language: Spanish. Translated by IDR]***

**STUDY ON CHILD AND YOUTH OBESITY**

**QUESTIONNAIRE**

**S1. For each minor between the ages 8 and 17 years, can you tell me the sex, month and year of birth, starting with the eldest? Please, tell me their first names so I can refer to them again later on:**

|  | Minors between 8 and 17 years | ME1 | ME2 | ME3 | ME4 | ME5 |
| --- | --- | --- | --- | --- | --- | --- |
| S1 | **First name** |  |  |  |  |  |
| S1.1 | **Sex:**  1. Male  2. Female | 1  2 | 1  2 | 1  2 | 1  2 | 1  2 |
| S1.2 | **What is their month and year of birth?** | /_/_/  Month  /_/_/_/_/  Year | /_/_/  Month  /_/_/_/_/  Year | /_/_/  Month  /_/_/_/_/  Year | /_/_/  Month  /_/_/_/_/  Year | /_/_/  Month  /_/_/_/_/  Year |

|  | Household members | MI1  Reference Minor | MI2  Informant | MI3 | MI4 | MI5 | MI6 |
| --- | --- | --- | --- | --- | --- | --- | --- |
| P1 | **What is their first name?** | /__/ | **/__/** | **/__/** | **/__/** | **/__/** | **/__/** |
| P2 | **Sex:**  1. Male  2. Female | 1  2 | 1  2 | 1  2 | 1  2 | 1  2 | 1  2 |
| P3 | **What is your/their month and year of birth?** | /_/_/  Month  /_/_/_/_/  Year | /_/_/  Month  /_/_/_/_/  Year | /_/_/  Month  /_/_/_/_/  Year | /_/_/  Month  /_/_/_/_/  Year | /_/_/  Month  /_/_/_/_/  Year | /_/_/  Month  /_/_/_/_/  Year |
| P4 | **What relationship do you/they have with**  1. Reference Minor  2. Mother  3. Father  4. Sibling  5. Uncle/Aunt  6. Grandparent  7. Other family member  8. Other person, not family (guardian, other) | 1 | 2  3  4  5  6  7  8 | 2  3  4  5  6  7  8 | 2  3  4  5  6  7  8 | 2  3  4  5  6  7  8 | 2  3  4  5  6  7  8 |
| P5 | **What is your/their current relationship status?**  1. Married or in de-facto relationship  2. Divorced  3. Separated  4. Single  5. Widowed  9. DK/NA |  | 1  2  3  4  5  9 | 1  2  3  4  5  9 | 1  2  3  4  5  9 | 1  2  3  4  5  9 | 1  2  3  4  5  9 |
| P6 | **What is the highest level of education achieved by?**  1. Can’t read or write  2. No education, but able to read and write  3. Primary (Until 5^th^ year EGB^[[1]](#footnote-1)^)  4. EGB completed (8^th^ year), or equivalent (elementary bachiller)  5. Professional Education^[[2]](#footnote-2)^ level1  6. Professional Education level2  7. Higher Secondary Education (BUP, Bachiller Superior^[[3]](#footnote-3)^)  8. University level, medium levels^[[4]](#footnote-4)^  9. University level, higher degree^[[5]](#footnote-5)^  10. Other, specify  99. DK/NA |  | 1  2  3  4  5  6  7  8  9  10  99 | 1  2  3  4  5  6  7  8  9  10  99 | 1  2  3  4  5  6  7  8  9  10  99 | 1  2  3  4  5  6  7  8  9  10  99 | 1  2  3  4  5  6  7  8  9  10  99 |
| P6B | **In what level are they currently enrolled?**  1. 1^st^ – Primary  2. 2^nd^ – Primary  3. 3^rd^ – Primary  4. 4^th^ – Primary  5. 5^th^ – Primary  6. 6^th^ – Primary  7. 1^st^ – Secondary  8. 2^nd^ – Secondary  9. 3^rd^ – Secondary  10. 1st Bachillerato  11. 2^nd^ Bachillerato  12. Professional Education  12. Other (specify)  14. Not studying  99. DK/NA | 1  2  3  4  5  6  7  8  9  10  11  12  13  14  99 |  |  |  |  |  |
| P7a | **What is the current employment status of?**  1. Employed  2. Unemployed, has previously been employed  3. Looking for first job  4. Retired (Previously employed)  5. Housewife/own work  6. Student  7. Disability / permanent disability  8. Other  9. DK/NA |  | 1  2  3  4  5  6  7  8  9 | 1  2  3  4  5  6  7  8  9 | 1  2  3  4  5  6  7  8  9 | 1  2  3  4  5  6  7  8  9 | 1  2  3  4  5  6  7  8  9 |

|  | Household Members | MI1  Reference Minor | MI2  Informant | MI3 | MI4 | MI5 | MI6 |
| --- | --- | --- | --- | --- | --- | --- | --- |
|  |  |  |  |  |  |  |  |
| P7c | **What is or was the main activity of the company where you/they work or the last company in which you/they worked?** |  | _________________________ | _________________________ | _________________________ | _________________________ | _________________________ |
| P7d | **What is the current occupation, or the last occupation held?** |  | _________________________ | _________________________ | _________________________ | _________________________ | _________________________ |
| P7e | **What is the professional situation that you/they have**  **or had in the company where you work or the**  **last professional category in which you/they have**  **worked?**  1. Self-employed without employees  2. Self-employed, with 10 or more  employees  3. Self-employed, with less than 10  employees  4. Manager of a company with 10 or more  employees  5. Manager of a company with less than 10  employees  6. Foreman, supervisor or manager  7. Another employee  9. No response |  | 1  2  3  4  5  6  7  8  9 | 1  2  3  4  5  6  7  8  9 | 1  2  3  4  5  6  7  8  9 | 1  2  3  4  5  6  7  8  9 | 1  2  3  4  5  6  7  8  9 |

**P8. In a normal week, how many days does he/she usually….**

|  |  | **No. of days per week.**  **From 0 to 7** |  |
| --- | --- | --- | --- |
| **P8.1** | Early Breakfast | **/__/** | DK/NA |
| **P8.2** | Snack or something to eat mid morning | **/__/** | DK/NA |
| **P8.3** | Main meal at midday/ Lunch | **/__/** | DK/NA |
| **P8.4** | Afternoon tea, or something to eat mid afternoon | **/__/** | DK/NA |
| **P8.5** | Dinner | **/__/** | DK/NA |
| **P8.6** | Something to eat after dinner | **/__/** | DK/NA |

**P9. Taking into account all the meals/snacks eaten, with what frequency does he/she eat the following foods per week?**

|  | Daily | A number of times per week | Occasionally | Never | DK/NA |
| --- | --- | --- | --- | --- | --- |
| 1. Fruit | 1 | 2 | 3 | 4 | 9 |
| 2. Vegetables | 1 | 2 | 3 | 4 | 9 |
| 3. White meat (poultry or rabbit) | 1 | 2 | 3 | 4 | 9 |
| 4. Red meat (Beef, pork, …) | 1 | 2 | 3 | 4 | 9 |
| 5. Fish | 1 | 2 | 3 | 4 | 9 |
| 6. Eggs | 1 | 2 | 3 | 4 | 9 |
| 7. Yogurt, | 1 | 2 | 3 | 4 | 9 |
| 8. Milk | 1 | 2 | 3 | 4 | 9 |
| 9. Bread, toast, bread roll | 1 | 2 | 3 | 4 | 9 |
| 10. Cereals | 1 | 2 | 3 | 4 | 9 |
| 11. Pasta or Rice | 1 | 2 | 3 | 4 | 9 |
| 12. Legumes | 1 | 2 | 3 | 4 | 9 |
| 13. Soup, casserole, stew | 1 | 2 | 3 | 4 | 9 |
| 14. Nuts | 1 | 2 | 3 | 4 | 9 |
| 15. Pizzas or Burgers | 1 | 2 | 3 | 4 | 9 |
| 16. Natural fruit juices | 1 | 2 | 3 | 4 | 9 |
| 17. Soft drinks | 1 | 2 | 3 | 4 | 9 |
| 18. Bottled juice | 1 | 2 | 3 | 4 | 9 |
| 19. Potato chips or snacks | 1 | 2 | 3 | 4 | 9 |
| 20. Chocolates, pastries | 1 | 2 | 3 | 4 | 9 |
| 21. Sweets, Candies | 1 | 2 | 3 | 4 | 9 |

**P10. On average, on a normal day, does he/she walk?**

|  | **P17.1** | **P17.2** | **P17.3** |
| --- | --- | --- | --- |
|  | **MI1 Reference Minor** | **Mother** | **Father** |
| 1. No. Never or almost never |  |  |  |
| 2. Yes, less than half an hour |  |  |  |
| 3. Yes, Around half an hour |  |  |  |
| 4. Yes, Between half an hour and an hour |  |  |  |
| 5. Yes, more than an hour |  |  |  |
| 9. DK/NA |  |  |  |

**P11. On a normal day, how many hours does he/she sleep?**

**/__/__/ hours**

**P12. On a normal day, does he/she usually watch television, use the computer, game console etc?**

1. Yes…………………. 🡪

2. No……………………🡪

9. DK/NA…………🡪

**P13. In general, do you think that having excessive weight can have negative health consequences for someone?**

1. No

2. Yes, although not as much as we get told by doctors, media etc

3. Yes, I think it is negative for health

9. DK/NA

|  |  | **MI1 Reference Minor** | **MI2 Informant** | **MI3** | **MI4** | **MI5** | **MI6** |
| --- | --- | --- | --- | --- | --- | --- | --- |
| **P13A** | **Declared weight (Kg)** | **/_/_/_/,/_/** | **/_/_/_/,/_/** | **/_/_/_/,/_/** | **/_/_/_/,/_/** | **/_/_/_/,/_/** | **/_/_/_/,/_/** |
| **P13B** | **Declared height (cm)** | **/_/_/_/** | **/_/_/_/** | **/_/_/_/** | **/_/_/_/** | **/_/_/_/** | **/_/_/_/** |
|  |  |  |  |  |  |  |  |
| **P13C** | **Measured weight (kg)** | **/_/_/_/,/_/** | **/_/_/_/,/_/** | **/_/_/_/,/_/** | **/_/_/_/,/_/** | **/_/_/_/,/_/** | **/_/_/_/,/_/** |
| **P13D** | **Measured height (cm)** | **/_/_/_/** | **/_/_/_/** | **/_/_/_/** | **/_/_/_/** | **/_/_/_/** | **/_/_/_/** |
|  |  |  |  |  |  |  |  |

**P14. Does he/she require some type of special commitment or care?**

|  | **MI1**  **Reference Minor** | **Mother** | **Father** |
| --- | --- | --- | --- |
| 1. Yes  2. No  8. Doesn’t know, not sure  9. No response |  |  |  |

**P15. How do you rate the security of the neighborhood where you live?**

1. Very good

2. Good

3. Average

4. Bad

5. Very bad

9. DK/NA

**P16. In general, the environmental quality of your neighborhood is….**

1. Very good

2. Good

3. Average

4. Bad

5. Very bad

9. DK/NA

1. Translation notes:

   EGB – General Education [↑](#footnote-ref-1)
2. Vocational Training, after General Secondary Education [↑](#footnote-ref-2)
3. Advanced School Leaving Exam [↑](#footnote-ref-3)
4. University qualifications of 1 cycle. E.g. University Diplomas and equivalent [↑](#footnote-ref-4)
5. University 2nd and 3rd cycle studies. E.g. University graduate and post graduate degrees [↑](#footnote-ref-5)
